# Cochlear Implantation after Bacterial Meningitis in Infants Younger Than 9 Months

**DOI:** 10.1155/2011/845879

**Published:** 2011-12-20

**Authors:** B. Y. Roukema, M. C. Van Loon, C. Smits, C. F. Smit, S. T. Goverts, P. Merkus, E. F. Hensen

**Affiliations:** ^1^Department of Otolaryngology and Head and Neck Surgery, VU University Medical Center Amsterdam, KNO ZH1D-116, P.O. Box 7057, 1007 MB Amsterdam, The Netherlands; ^2^Department of Audiology, VU University Medical Center Amsterdam, P.O. Box 7057, 1007 MB Amsterdam, The Netherlands

## Abstract

*Objective*. To describe the audiological, anesthesiological, and surgical key points of cochlear implantation after bacterial meningitis in very young infants. *Material and Methods*. Between 2005 and 2010, 4 patients received 7 cochlear implants before the age of 9 months (range 4–8 months) because of profound hearing loss after pneumococcal meningitis. *Results*. Full electrode insertions were achieved in all operated ears. The audiological and linguistic outcome varied considerably, with categories of auditory performance (CAP) scores between 3 and 6, and speech intelligibility rating (SIR) scores between 0 and 5. The audiological, anesthesiological, and surgical issues that apply in this patient group are discussed. *Conclusion*. Cochlear implantation in very young postmeningitic infants is challenging due to their young age, sequelae of meningitis, and the risk of cochlear obliteration. A swift diagnostic workup is essential, specific audiological, anesthesiological, and surgical considerations apply, and the outcome is variable even in successful implantations.

## 1. Introduction

Current standards for cochlear implantation in infants with severe congenital sensorineural hearing loss (SNHL) advocate an age at implantation between 9 and 12 months. On the one hand, a growing body of evidence indicates that hearing rehabilitation is more effective when the patient is implanted at a young age [1–4]. On the other hand, a certain period of time is needed to determine a reliable hearing threshold, to allow for improvement of hearing due to maturation of the auditory system after birth, and to test the performance of the patient with hearing aids [5]. Furthermore, the benefits of cochlear implantation before the age of 9 months should be weighed against the higher risk of anesthesia at this young age [5]. In case of sensorineural hearing loss caused by acute bacterial meningitis, different considerations apply. A swift diagnostic workup is imperative because of the risk of cochlear fibrosis and subsequent obliteration of the cochlear lumen, which may occur within weeks after the onset of meningitis, especially if the meningitis is caused by pneumococci [6, 7]. This diagnostic workup should include a thorough evaluation of the hearing as well as adequate imaging of the cochlea in order to assess the need and feasibility of cochlear implantation. In infants that suffer from postmeningitic SNHL, this may lead to an indication for cochlear implantation at an age younger than 9 months. If so, this patient group presents the cochlear implant (CI) team with a very specific set of challenges due to the young age of the patient, the additional sequelae of meningitis, and limitations to the time interval between the onset of meningitis and cochlear implantation. In order to illustrate these issues and discuss possible solutions and outcome, we describe our experience with patients that underwent cochlear implantation before the age of 9 months because of postmeningitic profound hearing loss. Furthermore, the specific diagnostic, anesthesiological, and surgical issues that have to be taken into consideration when performing cochlear implantations in very young postmeningitic patients are discussed.

## 2. Patients and Methods

We evaluated the patients younger than 9 months, who were selected for CI because of profound postmeningitic SNHL in the period from February, 2005 till March, 2010 at the VU University Medical Center, Amsterdam, The Netherlands.

All patients had participated in the Dutch youth health care programme. This programme is offered to all newborn children in The Netherlands and comprises of regular checkups (at the age of 2, 4, 8, 12, 16, 24, 30, and 48 weeks within the first year of age) by specialized physicians and youth health care workers, evaluating the physical health, immunology status, motor skills, speech functions, and the social, emotional, and psychological development of the infant. In the course of this programme, all four patients had received vaccines against *Streptococcus pneumoniae*, *Haemophilus influenzae* and *Neisseria meningitidis*. All patients had shown a normal development prior to the onset of meningitis.

In all patients, a full neurological and otolaryngological evaluation was performed. The causative microorganism was determined by culture of the cerebrospinal fluid. The audiological evaluation consisted of auditory brainstem response audiometry (ABR) and otoacoustic emissions (OAE) if possible in combination with visual reinforcement audiometry (VRA) or behavioral observation audiometry (BOA). In addition, all patients underwent a radiological evaluation consisting of high-resolution computed tomography (HRCT) of the middle ear and mastoid, and magnetic resonance imaging (MRI) of the brain and inner ear, including contrast-enhanced T1 weighted images and T2 weighted constructive interference steady state (CISS) images of the cochlea.

All patients were implanted with a Nucleus Freedom with Contour Advance electrode (C124RE (CA), Cochlear limited, Australia). The auditory and linguistic performance was evaluated 1 year after cochlear implantation. Parts of this evaluation are presented in Table 1, the Dutch version of the categories of auditory performance (CAP-NL) and the Speech Intelligibility rating (SIR) are presented in Tables 2 and 3, respectively [9, 10].

## 3. Results

Between 2005 and 2010, a total of 55 children were fitted with CI at our institution, 4 of which received the CI before 9 months of age because of bilateral severe SNHL caused by bacterial meningitis. All 4 patients were male. The youngest patient, aged 4 months at the time of implantation, was born prematurely at 33 weeks and 5 days gestation. He developed meningitis when he was 3 months of age and the other patients contracted meningitis at 5, 6, and 7 months of age (Table 1). Evaluation with ABR showed bilateral thresholds exceeding 85 dB in all patients but one. In this patient (case 2), ABR showed a hearing threshold exceeding 85 dB on the right side and a medium sloping SNHL (60 dB at 3 kHz) on the left (Table 1).

In all four cases, the meningitis was caused by *Streptococcus pneumoniae* even though they had all received a pneumococcal 7-valent vaccine (Prevenar, Pfizer) before the age of 5 months. All patients had a normal physical and psychological development at the time of the onset of meningitis.

**Figure 1:**
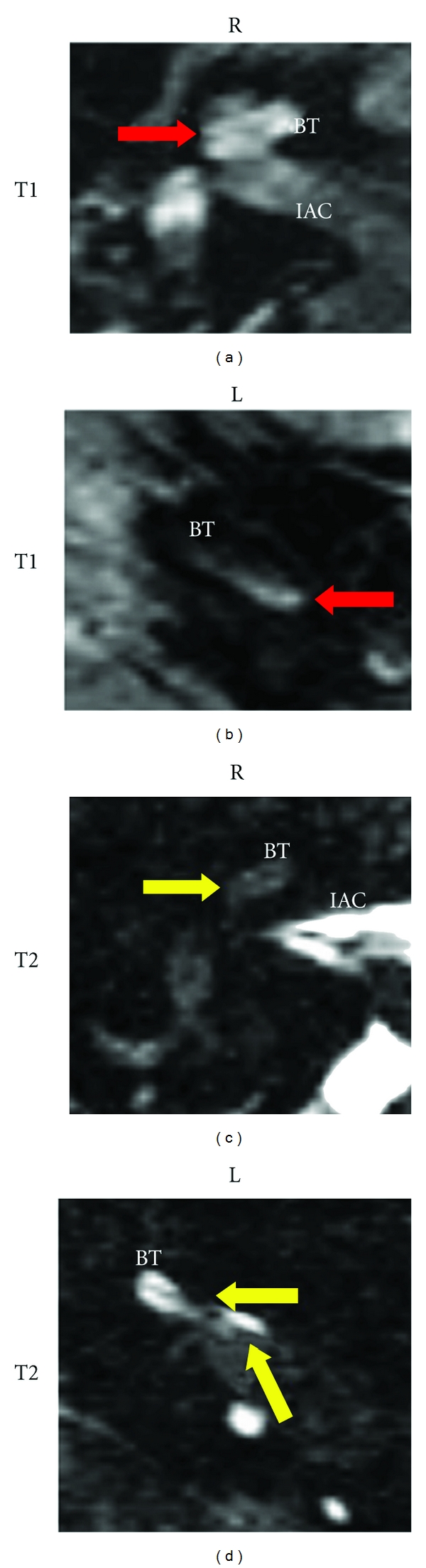
MR images of the right (R) and left (L) inner ears of a patient (case 2) after pneumococcal meningitis. Depicted are the axial T1 weighted MR images with contrast enhancement (T1, top row) and the T2 weighted MR images (T2, bottom row). The patient, a boy aged 7 months, suffered from asymmetric hearing loss after pneumococcal meningitis. Auditory brain stem response (ABR) audiometry showed a deaf ear on the right side and a sloping hearing loss (60 dB at 3 KHz) on the left side. Red arrows show contrast enhancement in the cochlea on the T1 weighted images of both ears ((a) and (b)). The contrast enhancement involves the whole cochlea and vestibulum on the right side, but it is limited to the basal turn (BT) on the left. Yellow arrows show loss of fluid in the cochlea on the T2 weighted images on both sides ((c) and (d)). Whereas on the right side, the loss of fluid involves the complete cochlea and the basal turn is barely visible, the loss of fluid only partially involves the basal turn of the left cochlea. IAC: internal auditory canal.

In accordance with the Dutch Consensus Protocol on Postmeningitic Hearing Evaluation, MR imaging was performed within 14 days after the identification of severe SNHL by ABR [7]. All cases showed enhancement of the cochlea on contrast enhanced T1 images, indicating active inflammation of the cochlea (Table 1). In the patient with asymmetric hearing loss (case 2), the best hearing ear (left side) showed enhancement of the scala tympani close to the round window in the basal turn only and no enhancement of the apical turn (Figure 1). T2 weighted images displayed a variety of outcomes in this patient group, varying from a hyperintense image indicating a normal fluid-filled cochlea, to a severe hypointense image, correlating with the formation of fibrous tissue or ossification within the cochlea (Table 1 and Figure 1) [11].

 Three patients received bilateral cochlear implants; one patient (case 2) with residual hearing at the left ear received a cochlear implant in the right ear and a hearing aid on the left side. The mean age at implantation was 6.5 months (range 4–8 months) (Table 1). All patients were implanted within a month of the diagnosis of SNHL (range 15–31 days). Peroperative findings included thickened perilymphe and minimal cochlear fibrosis in case 1 to more extensive cochlear fibrosis in cases 2, 3, and 4. We encountered no cochlear ossification, and full insertions were achieved in all operated ears (*n* = 7). There were no complications related to the surgery or CI activation. The key points of the anesthetical and surgical technique that have to be considered in this patient group are discussed below. The specific surgical issues are summarized in Table 4.

The auditory and linguistic outcome after cochlear implantation is summarized in Table 1. One year after implantation, we found considerable variation of the auditory performance within our patient group although all patients seem to benefit from the CI. The patient with the best performance (case 2), who had open set speech perception, was able to understand conversations without the aid of lip reading, and his speech was intelligible to all. The patient with the least favorable outcome (case 3) received bilateral implants at the age of 7 months and recognized sounds 1 year after implantation but was not able to understand words and had no intelligible speech. While in case 2, there appear to be no other meningitis-related sequelae beside the loss of hearing, case 3 also developed epilepsy, areflexia, cerebellar ataxia, and a developmental delay in cognitive and motor skills (Table 1).

## 4. Discussion

The young infant with profound SNHL due to bacterial meningitis presents specific challenges to the cochlear implant team. First, the time frame in this patient group is very different from congenitally deaf infants. In the latter, the currently reported optimal age at implantation is between 9 and 12 months of age, leaving ample time for extensive assessment of hearing, evaluation of possible improvement of hearing thresholds due to neuronal development after birth, a trial with hearing aids, cochlear imaging and the comprehensive counseling of parents. In postmeningitic profound SNHL, the risk of impending cochlear fibrosis and ossification resulting in increased surgical difficulty and risk of partial electrode insertion requires a swift audiological and radiological assessment and may necessitate cochlear implantation in infants younger than 9 months of age.

### 4.1. Sensorineural Hearing Loss after Bacterial Meningitis

Bacterial meningitis is the most common etiology for acquired hearing loss in children [12, 13]. Five to 35% of the patients with bacterial meningitis will develop permanent SNHL, which is profound and bilateral in up to 4% [14, 15]. Almost all bacteria species causing meningitis have been associated with permanent postmeningitic hearing loss, but this complication is most frequently found in *S. pneumoniae, N. meningitides, and H. Influenza* infections [6, 15, 16]. The prevalence of meningitis caused by these bacteria has decreased after the implementation of vaccination programmes in western countries [15, 17, 18]. The patients described in the current study also received vaccines against *S. pneumoniae, N. meningitides, and H. Influenza.* Even so, they all developed pneumococcal meningitis. Since 2006, all infants in The Netherlands are offered a pneumococcal 7-valent vaccine (Prevenar, Pfizer). Although this has led to a reduction in severe pneumococcal infections of approximately 50%, meningitis due to *Streptococcus pneumoniae *continues to occur (source: http://www.rivm.nl/). In The Netherlands, a new 10-valent vaccine (Synflorix, GSK) will replace the currently used 7-valent vaccine in 2011 because of the improved serotype immunization.

A loss of hearing caused by meningitis is not always readily apparent, especially in young infants due to their inability to communicate the problem and the possible cognitive effects of the infection. If SNHL remains undetected for a long period of time, it may critically affect the auditory and linguistic development [12, 15, 19, 20]. A formal audiological assessment is therefore mandatory in order to adequately identify the children at risk and prevent developmental delay due to missed SNHL [7]. The audiological evaluation should ideally be performed as soon as the medical condition of the patient allows, because cochlear ossification, resulting in increased risk of partial insertion of the CI electrode and a less favorable outcome, may occur as early as 3-4 weeks after the onset of meningitis [6, 7, 21–26]. Cochlear ossification is a known complication of *S. pneumoniae*, *N. meningitides*, and *H. influenza* infections, but pneumococci present the highest risk [6, 27].

### 4.2. Radiology and Decision Making

Profound SNHL after meningitis warrants a radiological evaluation of the temporal bone and cochlea ideally within 2 weeks of audiological assessment because of the risk of cochlear fibrosis and ossification (as discussed above) [7]. HRCT is an excellent tool for the evaluation of the temporal bone anatomy, but it is not suitable for the detection of cochlear fibrosis and its sensitivity for the detection of cochlear ossification is poor (40%) [6]. T2 weighted MR images (especially those with steady state sequence protocols such as CISS or FIESTA) are superior in the evaluation of the cochlear patency. Loss of fluid, seen as loss of the hyperintense signal in the cochlea, is evidence of fibrosis or ossification (Figure 1). T1 weighted contrast-enhanced MR images are useful in the identification of active cochlear inflammation, which is seen as contrast-enhancement within the cochlea. There is evidence that abnormalities on T1 contrast enhanced images precede loss of cochlear patency as seen on T2 images and that positive contrast enhancement is correlated with the occurrence of SNHL, accurately predicting a deterioration of sensorineural hearing after meningitis [28]. In line with this observation, we found contrast enhancement in all patients, but T2 abnormalities were only seen in case 2 (unilateral), 3 (bilateral), and 4 (bilateral). In case 2, the patent contralateral cochlea did show contrast-enhancement limited to the basal turn on T1 weighted images. The hearing in this ear was only partially affected and remained stable (a hearing threshold of 60 dB at 3000 Hz). We consider patients with bilateral profound hearing loss in combination with loss of cochlear patency as seen on T2 weighted MR images and/or active cochlear inflammation as identified on contrast enhanced T1 weighted MR images definite candidates for CI and would schedule the cochlear implantation as soon as their medical condition allows. In patients with unilateral hearing loss, MRI abnormalities in the best hearing ear warrant intensive audiological followup and cochlear implantation as soon as the hearing decreases.

### 4.3. Audiological Assessment and Counseling

The preoperative audiological evaluation and workup of young children with profound hearing loss after meningitis differs from other hearing impaired children, mainly because of the short time interval between assessing loss of hearing and cochlear implantation. Even so, thorough audiological assessment is essential in order to avoid unnecessary implantations. Ideally, a combination of objective measurements (ABR and OAE) and observational audiometry (BOA or VRA) should be performed [29]. However, in infants younger than six months, behavioral measurements cannot be used to reliably obtain hearing thresholds. Furthermore, the medical condition of the patient or the sequelae of meningitis may hamper behavioral observations. In addition, a trial with hearing aids, considered a standard procedure in most cochlear implant centers, is omitted if the MR imaging of the inner ear shows abnormalities indicative of inflammation or obliteration of the cochlear lumen following meningitis. The methods used for the hearing evaluation in very young postmeningitic CI-candidates therefore depend on the developmental age of the infant and its ability to cooperate. The audiological evaluation should at least include multiple objective measurements. Auditory brainstem response audiometry (ABR) is a well-established method to predict the hearing threshold around 2 to 4 kHz although the ABR response is not fully matured in infants younger than 6 months of age [30]. In some cases, more frequency-specific information is needed. For instance, children with moderate-to-severe hearing losses in the lower and middle frequencies and hearing loss exceeding 100 dB in the higher frequencies may show an absent click-ABR [31]. These children could greatly benefit from hearing aids and are not cochlear implant candidates per se. Other objective measurements like auditory steady state responses (ASSR), tone burst ABR, and electrocochleography may provide better frequency-specific information [32, 33].

In the short and often stressful period between the onset of meningitis, the recognition of profound SNHL and cochlear implantation, the parents need to be counseled, both on the fact that hearing loss has occurred as a complication of meningitis as well as on the benefits and risk of cochlear implantation. It is important that parents fully realize the fact that the hearing loss is profound and almost always permanent. In this process, behavioral observation audiometry may be helpful. As the expectations of cochlear implantation may be lower in postmeningitic CI candidates (see below), discussing realistic expectations is essential.

### 4.4. Anesthesiological Technique

Patients younger than 9 months of age have specific physiological characteristics that increase the risk of general anesthesia, and complications of meningitis may confer an even higher anesthetic risk. Specialized pediatric anesthesiologists are therefore an indispensable part of the pediatric cochlear implant team [5, 34]. Key points in the anesthesiological technique include the parental presence at induction, which significantly reduces separation anxiety and distress in the infant [35]. Gaseous or intravenous induction are both suitable, and the choice of anesthetic agent should be based on minimizing postoperative nausea and vomiting and minimizing the intraoperative bleeding. The use of facial nerve monitoring is strongly recommended but precludes the use of long-acting muscle relaxants. Special care must be taken with the positioning of the child. Because of the length of the procedure, wiring under the child or folds in clothes and draping can cause skin injury. It is important to minimize heat loss, as infants are particularly vulnerable to hypothermia because of a large body-surface-to-weight ratio [5]. The operation theatre should therefore be preheated, and a temperature control blanket should be applied. Conversely, prolonged surgery in a small surgical field using draping that covers a large surface area could increase the body temperature, and the body temperature should thus be monitored during the procedure [5]. If bilateral implantations are performed, the alternating position of the head should be anticipated. Furthermore, the pediatric trachea is of a shorter length, which makes the infant patient more prone to accidental extubation with head movement. Infants have higher relative oxygen consumption, and respiratory insufficiency due to suboptimal ventilation may rapidly escalate into a critical situation. Because of this, the tube should always be secured, preferably manually while positioning the head, and the anesthetist should be an expert in pediatric airway management [5].

Due to the small circulating blood volume, young infants are vulnerable to cardiovascular compromise, and meticulous hemostasis is of utmost importance. Hypovolemic effects can occur when blood loss exceeds 10% of the total blood volume [36]. This equals 65 mL of blood loss in a baby of 6 months (with an approximate weight of 8 kg) [5, 36]. The margin of safety in an infant of 4 months is obviously lower.

### 4.5. Surgical Technique

The specific surgical considerations in cochlear implantation in very young postmeningitic patients are summarized in Table 4.

**Figure 2:**
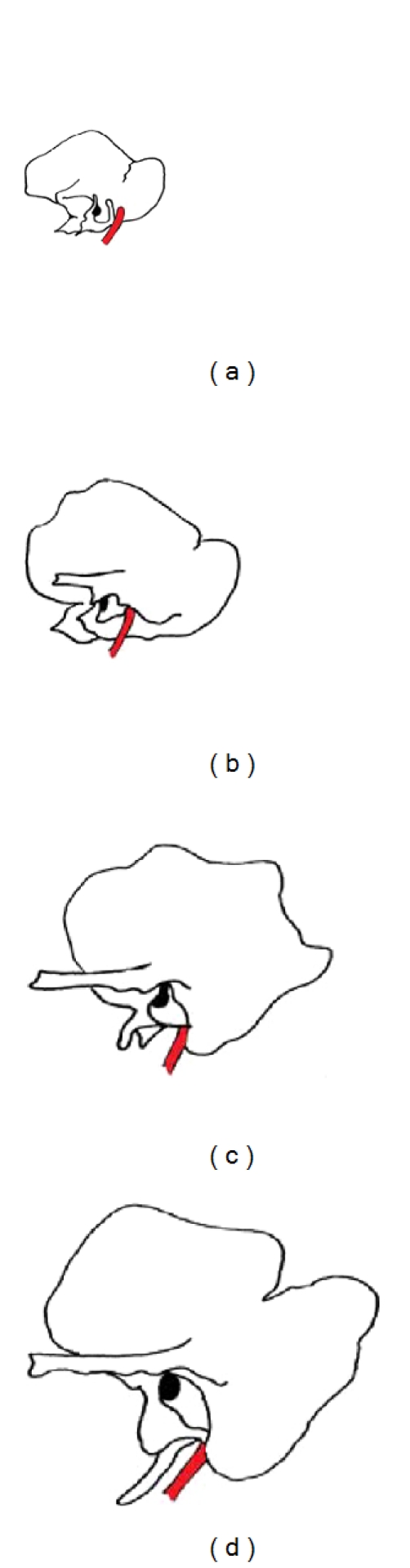
Development of the mastoid process. Schematic representations of the development of the temporal bone from infancy to adulthood (from (a) to (b)). In the young infant, the mastoid is small, and the facial nerve, marked in red, is not yet covered by the mastoid process.

**Figure 3:**
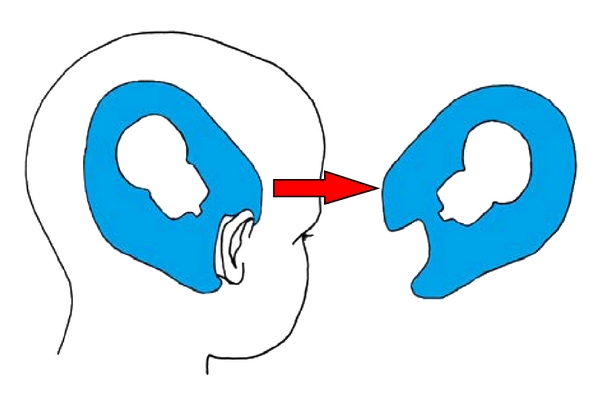
Drawing of a paper blueprint of the position of the implant relative to the ear in order to determine the correct, symmetrical position of the contralateral implant in bilateral implantation. The position of the implant at the first operated ear is marked on a paper sheet and transposed on to the contralateral side.

We perform a retroauricular S shape incision (“lazy S”), which allows for adequate exposure of the mastoid. It should not be extended downwards over the mastoid tip as far as in adults, because the undeveloped mastoid tip at this age does not yet cover the facial nerve, which is situated more superficial to the skin (Figure 2). When performing a bilateral implantation, symmetry must be observed in the placement of the implant. This can be achieved by creating a paper blueprint, marking the place of the implant relative to the ear, and using it to determine the correct position of the implant on the contralateral side (Figure 3). In order to avoid formation of a subcutaneous hematoma during bilateral surgery, a drain is placed lateral to the closed musculoperiosteal layer at the side of the first implanted ear. It can be taken out once the head bandage is in place.

Mastoid cells in very young children are relatively poorly pneumatized and contain bone marrow, causing profuse bleeding when performing the mastoidectomy [5, 37]. Hemostasis is important for an adequate surgical view but also because the small circulating blood volume of the infant does not allow for extensive blood loss [4]. As bipolar cauterization is often not helpful in this situation, hemostasis can be achieved by using diamond burrs and bone wax to obliterate the bleeding mastoid cells. Although the infant mastoid is small and sometimes consists of only the antrum, there is enough space for an adequate mastoidectomy and posterior tympanotomy [37]. The view through the posterior tympanotomy can be limited, however, due to the undeveloped mastoid and the restrictions in the angle looking through the posterior tympanotomy. In addition, the round window is often located in a more horizontal plane, parallel to the surgeons view.

Performing a cochleostomy can be a challenge in postmeningitic cases because of ossification of the cochlea. Even in cases with limited ossification, identification of the proper lumen is sometimes only possible after drilling out sections of the basal turn of the cochlea [25, 38]. Cochlear fibrosis or ossification may prevent full electrode insertions [6, 39, 40]. In some cases, a scala tympani insertion is impossible, and the electrode can only be placed in the scala vestibuli [41, 42]. Another solution may be a split electrode insertion [38–40]. In our patients, we did not encounter cochlear ossification, probably due to the short time interval that had elapsed between the onset of meningitis and cochlear implantation. We did, however, find cochlear fibrosis in case 2, 3, and 4, which could be overcome by gently removing it from the basal turn and subsequently inserting the electrode.

**Figure 4:**
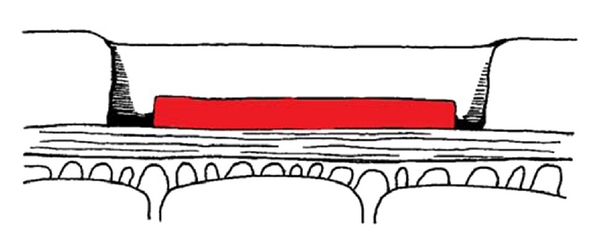
Schematic drawing of the construction of a bony island (in red). The cortical bone is thinned in the middle of the CI-shaped well, and the dura is completely uncovered at the borders of this well, creating an “island” of cortical bone protecting the dura.

When creating the bone bed for the cochlear implant, the thin cortex of the skull has to be taken into account. We perform a “bony island” construction, as it fixes the implant and minimizes the force on the skin and dura (Figure 4) [5, 37]. Alternatively, one may create a subperiosteal pocket only and avoid drilling a cortical well; however, this may affect the fixation of the implant in its position on the infant skull unless additional tie-down ligatures are placed [37].

**Figure 5:**
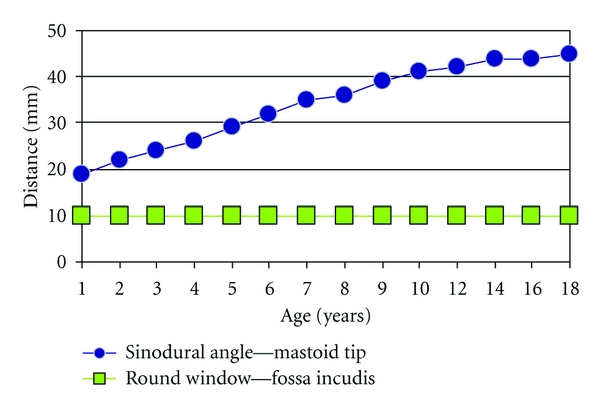
Growth of the middle ear versus mastoid: the mastoid tip develops, whereas the middle ear dimensions remain the same. The distance of the round window to the fossa incudis and facial recess does not change over time, but the mastoid process increases in size. When the electrode is fixed to the mastoid tip, the increasing distance from round window to mastoid tip could cause a possible displacement of the electrode out of the cochlea. Adapted from Dahm et al. [8].

 Finally, when fixing the electrode within the mastoid cavity, the altering dimensions of the developing temporal bone have to be taken into account. In contrast to the cochlea, the mastoid process is not fully developed at birth, and it expands during childhood (Figure 2). In the review of the growth pattern of the temporal bone by Dahm et al., it is demonstrated that whereas the distance between the round window and the fossa incudis does not increase after birth, the distance between the round window and the sinodural angle as well as the distance between the fossa incudis and the mastoid tip increase considerably during the first 18 years of life (Figures 2 and 5) [8]. Fixation of the lead on the electrode in the caudal part of the mastoid is therefore not advisable, as the development of the mastoid tip could cause dislocation of the electrode. In addition, there has to be enough lead (about 20–25 mm) on the electrode to allow for the increase in distance between the round window and the implant fixed to the skull. Fixation of the electrode at the round window or cochleostomy and of the electrode lead within the posterior tympanotomy is safe and will support a proper electrode position during childhood. If these surgical considerations are taken into account, cochlear implantation in very young children is not associated with an increased risk of surgical complications [37, 39].

### 4.6. Outcome

The outcome of cochlear implantation in postmeningitic infants is less predictable than the outcome in congenitally deaf children [6, 39]. It is not only dependent on the proper CI placement and the depth of electrode insertion, which can be compromised in these patients due to obliteration of the cochlear lumen, but also on the type and severity of additional sequelae of meningitis if present. Bacterial meningitis may cause damage to the cochlear spiral ganglia, which may result in failure of the neuronal response even in cases with full electrode insertions [43, 44]. Moreover, the outcome of cochlear implantation also depends on the cognitive and linguistic abilities of the recipient, which is of special significance in patients with profound SNHL due to meningitis, as this condition may affect these factors as well. This is also reflected in the considerable variation in audiological performances of our patient group, ranging from open set speech perception to the identification of sounds only (Table 1). Not surprisingly, the best performing patient (case 2) had no other complaints besides hearing loss, whereas the patient with the worst performance (case 3) suffered from severe neurological sequelae (Table 1). Importantly, postmeningitic children seem to benefit from CI even in case of incomplete insertions or comorbidity associated with meningitis [42].

## 5. Conclusion

Cochlear implantation is indicated in infants younger than 9 months if postponing surgery would decrease the chances of successful implantation. This is the case in profound SNHL and impending obliteration of the cochlear lumen due to fibrosis or ossification caused by meningitis. In postmeningitic patients younger than 9 months, cochlear implantation is feasible, but specific diagnostic, anesthesiological, and surgical considerations related to the early age at implantation and the possible sequelae of bacterial meningitis apply. Furthermore, the outcome of CI in postmeningitic infants is variable even in technically successful implantations. A multidisciplinary CI team, consisting of pediatric audiology, anesthesia, speech therapy, and otology specialists is therefore essential in the successful management of this challenging patient group.

## Figures and Tables

**Table 1:** Clinical characteristics and outcome of infants receiving cochlear implantation because of postmeningitic profound sensorineural hearing loss before the age of 9 months.

Case	Age at onset meningitis	ABR results	T1+ contrast cochlear MR image	T2 weighted cochlear MR image	Age at cochlear implantation	Side of implantation	Surgical findings	Result of implantation	Categories of auditory performance (CAP-NL)	Speech intelligibility rating (SIR)	Other sequelea
1	3 months	>85 dB L + R	enhancement cochlea L + R	normal hyperintense	4 months	L + R	minimal cochlear fibrosis	full insertions	4	0	epilepsy
2	5 months	>85 dB R 60 dB L	enhancement cochlea L + R	unilateral hypointensity	7 months	R	cochlear fibrosis	full insertion	5-6	5	none
3	6 months	>85 dB L + R	enhancement cochlea L + R	hypointensity and artifacts	7 months	L + R	cochlear fibrosis	full insertions	3-4	1	epilepsy, areflexia, ataxia, and developmental delay
4	7 months	>85 dB L + R	enhancement cochlea L + R	severe hypointense	8 months	L + R	cochlear fibrosis	full insertions	3	5	attention deficit, and hemiparesis of tongue

**Table 2:** The dutch categories of auditory performance (CAP-NL).

Categories of Auditory Performance (CAP-NL)	Score
Use of telephone with known speaker	7
Understanding of conversation	6
Understanding common phrases without lip-reading	5
Discrimination of speech sounds without lip-reading	4
Identification of environmental sounds	3
Response to speech sounds	2
Awareness of environmental sounds	1
No awareness of environmental sounds or voice	0

**Table 3:** Speech intelligibility rating (SIR) criteria.

Speech intelligibility rating (SIR)	Score
Connected speech is intelligible to all listeners. Child is understood easily in everyday context.	5
Connected speech is intelligible to a listener who has little experience of a deaf person's speech.	4
Connected speech is intelligible to a listener who concentrates and lip-reads.	3
Connected speech is unintelligible. Intelligible speech is developing in single words when context and lip reading cues are available.	2
Connected speech is unintelligible. Prerecognizable words in spoken language, primary mode of communication may be manual.	1

**Table 4:** Problem solving during cochlear implantation in postmeningitic infants.

Problem	When	Suggested technique
Superficial course of facial nerve	At incision	Less pressure on the knife and more superior incision.
Bilateral “symmetrical” position of the implant	At incision	Drawing of the position of the implant on a blueprint and copy at the contralateral side (Figure 3).
Profuse bleeding because of bone marrow filled mastoid	During mastoidectomy	Use diamond burrs and close off the mastoid cells with bone wax.
“Thick” implant and thin skull cortex	During creation of the implant bed	Create a bony island over the dura (Figure 4).
Round window in a more horizontal plane	Before cochleostomy	Make the posterior tympanotomy as wide as possible, and drill towards stapes to find round window.
Ossification of the cochlea	At cochleostomy and electrode insertion	Drill-out of basal turn of the cochlea, partial electrode insertion, scala vestibuli insertion, or split electrode insertion.
Hematoma at the first implanted ear	At closure of first side	Place surgical drain superficial of the musculoperiosteal flap, remove after head bandage.
Electrode can dislocate out of the cochlea	During development of the mastoid process	Position and fixation of the electrode lead in the round window, posterior tympanotomy, but not in the mastoid tip region. Ensure there is enough lead on the electrode to allow for development of temporal bone.
